# The Rationality of the Hypolipidemic Effect of *Alismatis Rhizoma* Decoction, a Classical Chinese Medicine Formula in High-Fat Diet-Induced Hyperlipidemic Mice

**Published:** 2014

**Authors:** Chengwu Song, Xiaofei Huang, Kungang Lu, Min Peng, Shanggong Yu, Nianbai Fang

**Affiliations:** *Key Laboratory of Chinese Medicine Resource and Compound Prescription (Hubei University of Chinese Medicine), Ministry of Education, 1 Huang-jia-hu, Wuhan, China.*

**Keywords:** *Alismatis Rhizoma* decoction, Hypolipidemic effect, Synergistic effects

## Abstract

*Alismatis Rhizoma* Decoction (ARD) is a classical Traditional Chinese Medicine (TCM) formula for treatment of vertigo with its long history of successful clinical effect. Since vertigo is a symptom of hyperlipidemia, this study aimed at evaluating the hypolipidemic effect of ARD in hyperlipidemic mice induced by high fat diet (HFD) and investigated the rationality of formula combination of *Alismatis Rhizoma* (AR) and *Atractylodis Macrocephalae Rhizoma* (AMR). Compared with control group, hyperlipidemic mice in AR and ARD groups displayed a reduction of the following parameters: body weight, liver and serum total cholesterol, triglyceride concentration, liver and spleen coefficients, activities of serum aspartate aminotransferase (AST) and alanine aminotransferase (ALT); whereas the serum HDL-cholesterol levels were significantly elevated in both AR and ARD groups. AR and ARD treatments significantly down regulated the expressions of 3-hydroxy-3-methylglutharyl-coenzyme A reductase (HMG-CoA reductase) and sterol regulatory element binding factor-2 (SREBF-2). These findings clearly provided evidences that the suppression on biosynthesis of cholesterol in liver may in part contribute to the hypolipidemic effects of ARD and AR. Since no significantly hypolipidemic effect of AMR was observed, the more prominent effect of ARD than that of AR indicated synergistic effects of AR and AMR, and confirmed the rationality of ARD formula.

## Introduction

Hypercholesterolaemia is considered as a risk factor involved in the development of cardiovascular disease, which is among the ten most common health problems in the world currently (1, 2). The search for hypolipidemic drugs resulted in numerous synthetic agents such as the inhibitors of HMG-CoA reductase with well lipid-lowering activities. However, most synthetic hypolipidemic drugs have adverse effects. For example, the adverse effect of statins, rhabdomyolysis, led to the voluntary withdrawal of cerivastatin (Baycol) by its manufacturer (3). TCM products are frequently considered to be less toxic than synthetic agents (4) and a good option for treatment of hypercholesterolaemia. TCMs are usually prescribed in combination of more than one TCM materials to obtain synergistic effect and diminish adverse drug reactions. It should be noted that the formula of ARD originated from the Jin Gui Yao Lve written by Zhongjing Zhang, a famous doctor of Han Dynasty (220 A.D.) in ancient China (5). ARD is composed of two TCM plant materials, AR and AMR. In TCM clinical practice, ARD is used to treat vertigo that is a symptom of hyperlipidemia patients (6). Chen *et al.* found the anti-hyperlipidemia effect of modified ARD formula was similar to that of Lovastatin on essential hyperlipidemia patients (7). The study of the anti-hyperlipidemia effect on obese rats indicated that ARD combined with Erchen Tang were higher potent on regulation of rat serum total cholesterol, triglyceride, HDL-cholesterol, LDL-cholesterol levels than Oenothera Biennis Oil (8). The hypolipidemic effect of another modified ARD formula was more apparent than the formula Captopril Tablets (9). However, little information is available about the rationality of ARD formula and the molecular mechanisms of its hypolipidemic effect.

The hypotensive effect of ARD and hypolipidemic effect of AR have been reported in our previous study (10, 11). Both of lipid metabolism disorders and hypertension often are associated with each other, sharing the congenerous metabolic abnormalities and genetic background; and they are reciprocal causation in a variety of mechanisms (12, 13). The present study was designed to evaluate the effects of ARD and its component TCMs AR and AMR on serum and liver lipids in mice fed HFD. The lipid profile was taken as the major marker of hypercholesterolaemia. Accordingly, serum total cholesterol (TC), triglyceride (TG), HDL-cholesterol (HDL-C), and liver TC, TG were among the parameters investigated. Meanwhile, the effects of ARD and its component TCMs AR and AMR on the liver pathological changes were also examined. Specifically, we assessed influence of ARD and its component TCMs AR and AMR on the three key hepatic cholesterol metabolism genes for HMG-CoA reductase, SREBF-2 and cholesterol-7alpha-hydroxylase (CYP7A1) in order to find the potential molecular mechanism by which ARD modulated lipid profiles. 

## Experimental

*Reagents and* *TCM materials*

Cholesterol and sodium cholate were purchased from Beijing Shuangxuan Microbe Culture Medium Products Factory (Beijing, China). The plant of AR and AMR were respectively grown in Sichuan and Zhejiang Province, China. AR (Batch number, 1012036) and AMR (Batch number, 1001022) were endotoxin-free and commercially provided by Hubei Kangjin Pharmaceutical Co*. Ltd*. (Xianning, Hubei, China). The herbs were identified and authenticated by the taxonomist of Key Laboratory of Chinese Medicine Resource and Compound Prescription (Hubei University of Chinese Medicine), Ministry of Education. Voucher specimens (No. 040 and 041) were deposited in the herbarium of Key Laboratory of Chinese Medicine Resource and Compound Prescription (Hubei University of Chinese Medicine), Ministry of Education. 

*Preparation of TCM extracts*

Same amount (2.8 Kg) of AR, AMR and ARD (mixture of AR:AMR in ratio 5:2, w/w), respectively, in 22,400 mL (2,800 g x 8 mL/g) of distilled water were decocted for 2 hours and filtered through a Büchner funnel with a no. 4 filter paper. The extraction process was repeated with same amount of distilled water for two times. The three extracts were combined and concentrated on a rotatory evaporator under reduced pressure followed by drying in a freeze-dryer. The dried extracts represented 11.33%, 60.2%, 21.57% of the original TCM materials AR, AMR, ARD, respectively. 

*Animals and treatments*

Male Kunming mice (18-22 g) were purchased (certificate No.: SCXK 2008-0003) from Wuhan institute of Biological Products (Wuhan, China). All animals were free access to a commercial diet (Wuhan institute of Biological Products, China) and water in an air-conditioning room with a 12:12 h light: dark cycle. Temperature and humidity were controlled at 23 ± 2 °C and 60% ± 5%, respectively. Mice were acclimatized for 3 days before being randomly divided into 2 groups. The first group had 8 mice and continued to receive a regular diet as the control group. The rest mice were transferred to HFD, which was made of commercial diet (78.8%), egg yolk (10%), lard (10%), cholesterol (1%) and cholate (0.2%) (14). Blood samples were collected from caudal vein of mice after feed of HFD for four weeks. The level of serum lipid was measured, and a significant increase of serum lipid level suggested an establishment of hyperlipidemia mouse model. 

Thirty-two hyperlipidemic mice were selected on the basis of body weight, levels of serum lipids and randomly divided into 4 subgroups. There were no significant differences between groups at the start of the experimental period. The extracts were homogenized in 0.5 mL of distilled water for one time intragastric administration. Mice in control (C) group and HFD group were intragastrically administered with 0.5 mL of distilled water one time per day. The remaining three groups (AR, AMR and ARD) were treated with 2.26 g Kg^-1^d^-1^ of AR extract, 12.04 g Kg^-1^d^-1^ of AMR extract and 4.31 g Kg^-1^d^-1^ of ARD extract, respectively. Each extract Kg^-1^d^-1^ was equivalent to 20.0 g TCM materials. The experimental groups mice were daily intragastrically administrated with TCM extracts once a day for 28 consecutive days. The mice in C group were maintained on a commercial diet, and other groups continued to be fed with HFD. The body weight and food consumption were recorded weekly. All animal experimental procedures were approved by the Institutional Animal Care and Use Committee of Hubei University of Chinese Medicine.

Blood samples (200 uL) were collected on days 0 and 14 through caudal vein from mice with fasting diets for 12 hours but free access to water. On the 28^th^ day, the third batch of blood samples were collected by ophthalmectomy after anesthetized with pentobarbital injection. The mice after blood collection were sacrificed and the livers, hearts, spleens, lungs and kidneys were quickly removed and weighed before freezing for storage at −80 °C. The sera separated by centrifugation and organs were stored at −80 °C until analysis. The organ coefficient was calculated as: organ weight/body weight at sacrifice x 100.

*Biochemical Analysis*

Serum TC, TG and HDL-C were analyzed using commercial reagent kits (Shanghai Mind Bioengineering Co. *Ltd*., Shanghai, China). Serum AST and ALT were determined by commercial reagent kits (Nanjing Jiancheng Bioengineering Institute, Nanjing, Jiangsu, China) in accordance with the manufacturer’s protocol. The lipids of liver tissue were extracted on the basis of published method with several modifications (15). Briefly, 200 mg of liver tissue was homogenized in dichloromethane-methanol (2:1, v/v). Sodium chloride was added to mixture and mixed. The mixture was centrifugated and aliquot of the organic phase was mixed with Triton X-100. After evaporation of the organic solvents, the TC and TG contents in sample were measured by the TC and TG Kits (Shanghai Mind Bioengineering Co. *Ltd*., Shanghai, China).

*Histological analysis*

Dissected liver samples were fixed with 4% paraformaldehyde in 0.1 M sodium phosphate buffer (pH 7.4) overnight at 4 °C. After rinsing with phosphate-buffered saline, the samples were dehydrated and embedded in paraffin wax. Paraffin sections were prepared using a microtome at a thickness of 4 μm, followed by hematoxylin and eosin (HE) staining. The histological changes of livers after treatments were observed at a light microscope.

*Analysis of liver mRNA*

Total RNA was isolated according to the manufacturer's recommended protocol using the Simply P Total RNA Extraction kit (Bioflux, Japan) for mRNA analysis. The quality of the total RNA isolated was assessed by examining RNA purity using spectrophotometer set at wavelengths of 260 and 280 nm, and estimating the 260:280 nm ratios (1.7-2.0). Isolated RNA was used to synthesize cDNA with a RevertAid^TM^ First Strand cDNA Synthesis Kit #k1622 (Fermantas, EU) according to the manufacturer's protocol. RT-PCR was carried out with the SsoFast^TM^ EvaGreen Supermix from Bio-Rad laboratories (Hercules, USA) on a Bio-Rad iCycler machine (California, USA). The pairs of forward and reverse primers were synthesized by Invitrogen-Life Technologies (Shang Hai, China) (Table 1). A housekeeping transcript, *β*-actin, was used as an internal control because of its stable expression *in-vivo* (16)*. *Product specificity was examined by dissociation curve analysis. The amplified gene (4.8 uL) was resolved using agarose gel electrophoresis under 100 V and was stained with GelRed^TM^. Analysis of the PCR products was carried out with the Launch Vision Works LS and the Gel Doc-IT^TM^ Imaging System. The level of mRNA was expressed as -fold induction to that of untreated control.

**Table 1:** Polymerase chain reaction primer sequences

**Mouse Gene**	**Primer**	**Sequence** **
β-Actin	Forward	CAC TgT gCC CAT CTA CgA
	Reverse	CAg gAT TCC ATA CCC AAg
CYP7A1*	Forward	AgT TAC TCT TCC CgT TTC
	Reverse	ATC ACC TCC AgC CTC TAC
SREBF-2*	Forward	AAATCCACGGTCCAAGCC
	Reverse	GTGCGTCTATCAAGTCCAGAAT
HMG-CR*	Forward	gTT CTT TCC gTg CTg TgT TCT ggA
	Reverse	CTg ATA TCT TTA gTg CAg AgT gTg gCA C

* CYP7A1, cholesterol-7alpha-hydroxylase; SREBF-2, sterol regulatory element binding factor-2; HMG-CR, 3-hydroxy-3-methylglutharyl-coenzyme A reductase.

** primers are shown 5’→3’.

*Statistical analysis*

Results are presented as the means + *SD*. The data were analyzed by one-way analysis of variance (LSD test) to compare treatments vs the control (HFD group) using SPSS 19.0 software. p*-*values < 0.05 were considered statistically significant.

## Results

*General parameters*

**Figure 1:**
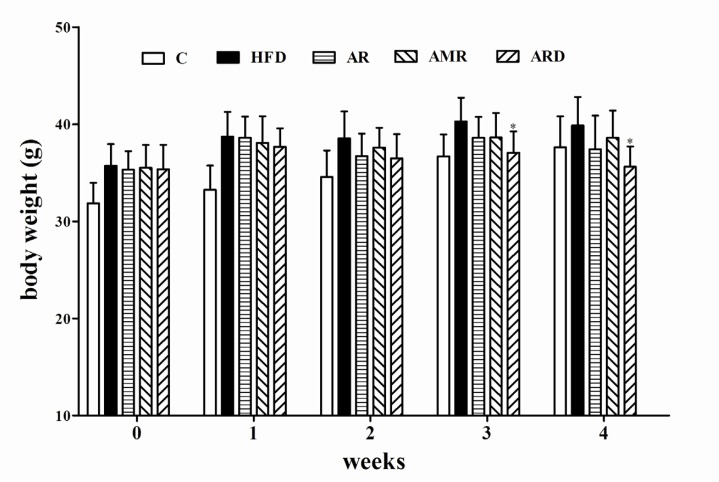
Values of body weight in mice treated with TCMs for four weeks. ^*^P<0.05 significantly different from samples in HFD group.

There were no significant differences in a diet intake among groups. As shown in Figure 1, the body weights of mice fed regular diet (C group) were significantly lower than that of mice in HFD group during 0-3^rd^ weeks. 

However, after 4 weeks extract treatments the body weights of mice in control group did not have significant difference from mice in all groups fed HFD. The body weight of mice treated with ARD had significantly lower body weights than that of mice in HFD group at the end of the experiment. In addition, the body weight of mice in AR and AMR slightly decreased compared to that of HFD group, however, the difference was not significant. Table 2 shows the liver and spleen coefficients significantly increased in hyperlipidemic mice compared to that of C group. ARD treatment significantly reduced these two coefficients in hyperlipidemic mice as compared with hyperlipidemic mice in HFD group. Meanwhile, these two coefficients slightly decreased in AR and AMR groups, however, the difference was not statistically significant. In addition, heart, lung and kidney coefficient values in all groups were not significantly influenced by HFD and TCM (AR, AMR and ARD) extract treatments during the study (data not shown).

**Table 2:** Values of organ coefficients and biochemical parameters in mice treated with TCMs (mean + SD, n=8)**.**

	**C**	**HFD**	**AR**	**AMR**	**ARD**
Liver coefficient	4.53 + 0.33	5.23 + 0.44	4.93 + 0.39	4.91 + 0.36	4.75 + 0.37*
spleen coefficient	1.44 + 0.27	2.37 + 0.32	2.07 + 0.26	2.14 + 0.24	1.95 + 0.25*
Serum ALT (U/mL)	52.14 + 6.15	92.54 + 8.14	75.19 + 7.13**	80.53 + 7.48*	70.46 + 6.72**
Serum AST (U/mL)	71.40 + 11.88	146.13 + 21.50	112.01 + 17.32**	120.93 + 21.73*	95.08 + 21.13**
Liver TC (mg/g)	3.93 + 0.50	40.35 + 5.91	30.78 + 4.52**	35.24 + 5.85	24.33 + 4.67**#
Liver TG (mg/g)	10.42 + 2.57	18.81 + 2.95	13.27 + 1.94**	16.15 + 2.54	12.51 + 1.48**

* *P<0.05*,

** *P<0.01* significantly different from samples in HFD group.

# *P<0.05* significantly different from samples in AR group.

*Biochemical* *parameters*

**Figure 2:**
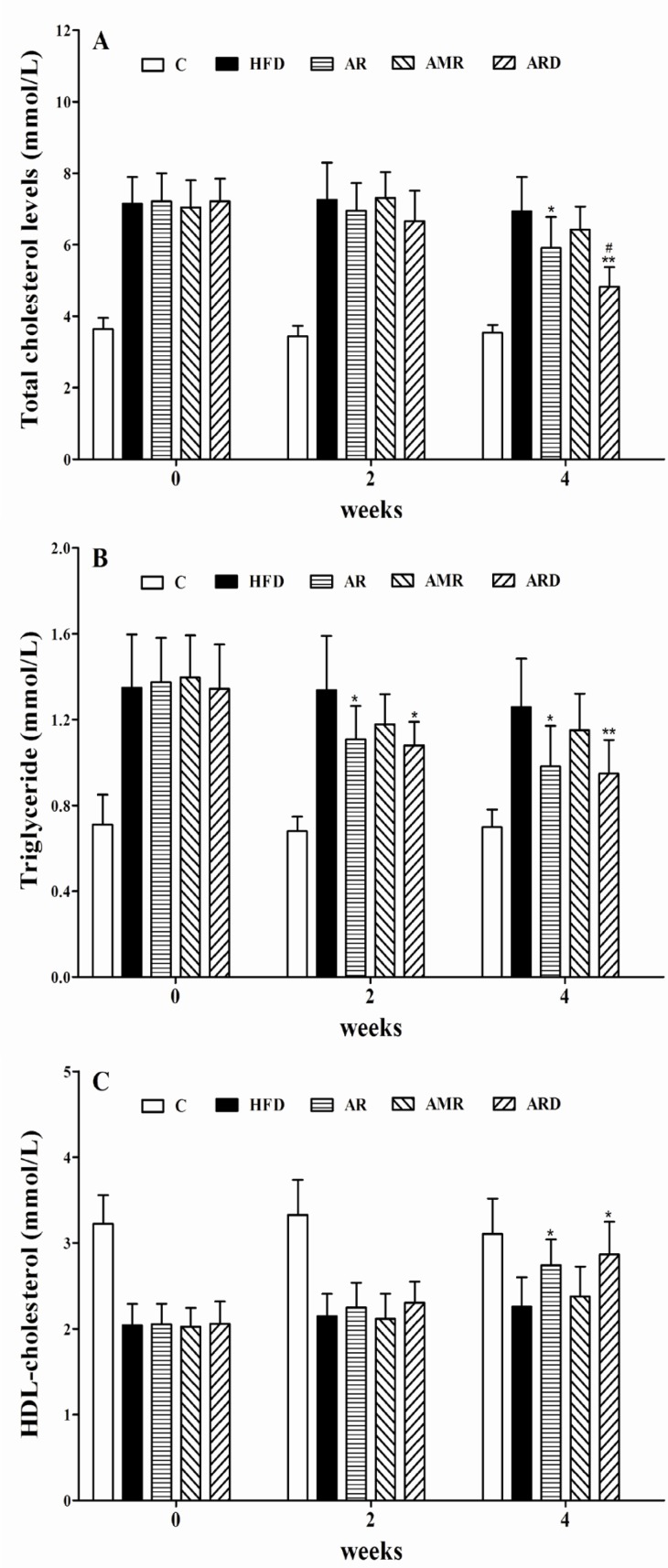
Values of serum TC (**A**), TG (B) and HDL-C (**C**) levels in mice treated with TCMs for four weeks. ^*^P<0.05, ^**^P<0.01 significantly different from samples in HFD group. ^#^*P<0.05* significantly different from samples in AR group.

The marked increase in levels of serum TC and TG indicated an establishment of a mouse model of hyperlipidemia induced by HFD (Figures 2A and 2B). After 4 weeks treatments, serum TC levels were 5.91 units for AR group, and 4.83 units for ARD group, which were 14% and 30% lower than 6.93 units of HFD group, respectively. Also, the observed decrease of TC level in ARD group was statistically significant, when compared to that of group AR. The serum TG levels after AR and ARD treatments were 0.98 and 0.94 units, which were 78% and 74% of 1.26 units in HFD group. As shown in Figure 2C, serum HDL-C level was significantly elevated in mice treated with AR and ARD in comparison with that of HFD group after 4 weeks’ treatments. However, the differences of serum TG and HDL-C between AR and ARD groups were not significant (Figure 2B, 2C). The levels of serum ALT activity were significantly decreased from 92.54 units in HFD group to 75.19, 80.53 and 70.46 units with AR, AMR and ARD treatments, respectively. Meanwhile, the levels of serum AST activity of mice treated with AR, AMR and ARD were 112.01, 120.93 and 95.08 units, which were significantly lower than that of HFD group (146.13 units) (Table 2). The levels of liver TC and TG were increased in HFD group whereas the increases were markedly improved in AR and ARD treated animals (Table 2). AR and ARD significantly decreased liver TG levels to 70% and 66% of HFD group at the end of 4 weeks treatment periods. Also, the significant mean decreases of liver TC level were 24% and 40% lower than that of HFD group. Furthermore, the liver TC levels in ARD group were the lowest in all treatment groups, and had a significant difference compared with AR group. 

*Histopathologic comparisons of livers*

**Figure 3:**
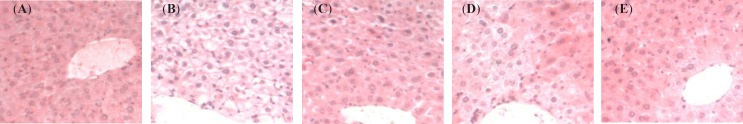
Representative light microscopic photographs of liver sections stained with HE in mice treated with TCMs for four weeks. A, C group; B, HFD group; C, AR group; D, AMR group and E, ARD group. a, large lipid droplets; b, eccentric nucleus; c, hepatocyte injury, ballooning degeneration and d, polymorphonuclear leukocyte infiltration. All images were photographed at 400 x magnification

Figure 3 shows comparative histopathology for livers of mice at the study termination point of 4 weeks. Histological examination of liver sections by HE staining revealed the structure of the liver tissue was intact, and the hepatocytes were radially arranged around the central vein only in the C group (Figure 3A). The liver of mice showed a loss of cellular integrity, marked accumulation of large lipid droplets (Figure 3B, a) and eccentric nucleus (Figure 3B, b) in the HFD group. Also, hepatocyte injury, ballooning degeneration (Figure 3B, c) and polymorphonuclear leukocyte (PMN) infiltration (Figure 3B, d) were seen in the liver of hyperlipidemic mice. However, the liver histopathologic changes were markedly improved in AR and ARD treated animals by the evidence of less large lipid droplets. 

*Expressions of hepatic* *cholesterol related enzymes* *mRNA*

**Figure 4:**
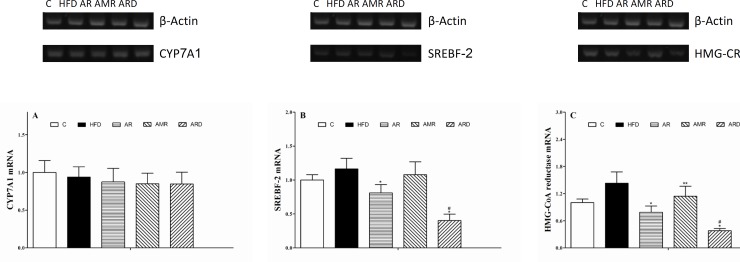
Three key hepatic cholesterol metabolism genes mRNA relative quantities in mice treated with TCMs for four weeks. **A**, CYP7A1; B, SREBF-2 and **C**, HMG-CoA reductase.^ *^P<0.01, ^**^P<0.05 significantly different from samples in HFD group. ^#^*P<0.05* significantly different from samples in AR group

The values of hepatic CYP7A1, SREBF-2 and HMG-CoA reductase mRNAs (Figure 4) in the mice livers were determined by normalizing them to the value of β-Actin mRNA. There were no significant differences in the relative quantities of hepatic CYP7A1 mRNA among all groups. The relative quantity of hepatic SREBF-2 mRNA in the ARD group tended to be the lowest in all of the groups. ARD treatment of hyperlipidemic mice induced 1-fold decrease of hepatic SREBF-2 mRNA by comparison with that in AR group. The relative quantities of hepatic HMG-CoA reductase mRNA in mice of AR, AMR and ARD groups were reduced to 55%, 80% and 27% of the values in HFD group. By comparison with AR group, the relative quantity of HMG-CoA reductase mRNA was significantly lower in ARD group. Meanwhile, the relative quantity of hepatic SREBF-2 mRNA in AMR group slightly decreased compared to that of HFD group, but the difference was not significant. 

## Discussion

The present study was to evaluate the hypolipidemic effects of ARD and its TCM components in hyperlipidemic mice, which were used to confirm the rationality of AR and AMR combination for ARD formula. Also, three key hepatic cholesterol metabolism genes for HMG-CoA reductase, SREBF-2 and CYP7A1 were assessed to find the potential molecular mechanism. As expected, male hyperlipidemic mice had significantly greater body weight, serum and liver cholesterol levels before TCM treatments. The pattern of body weight gain in mice may be due to accumulation of subcutaneous fat and visceral fat. The results revealed that AR and ARD significantly reduced the levels of serum and liver cholesterol in hyperlipidemic mice after 4 weeks treatments. Cholesterol reduction could be achieved by reducing plasma cholesterol, decreasing hepatic synthesis, or enhancing cholesterol degradation and excretion (16). Cholesterol concentration in the blood is affected by both cholesterol content of diet and cholesterol synthesized in liver. The intake of diets did not have any difference among each group during the whole experiment, which eliminated the influence of exogenous cholesterol. Therefore, it is reasonable to assume that the decline of the serum cholesterol concentrations in the mice of AR and ARD groups may be attributed to the decreasing of endogenous cholesterol contents. This hypothesis was supported in part by the facts that increase of serum HDL-C level was accompanied with decrease of serum TC level in mice (Figure 2). The concentration of serum HDL-C is a major indicator to evaluate the potent of HDL facilitating the translocation of cholesterol from the peripheral tissues to liver for catabolism (17). In the present study, the HFD greatly increased fat storage with a consequence of 2-fold higher serum AST levels in hyperlipidemic mice (HFD group) than that in C group, which led to severe liver tissue damage as shown in the histological analysis. The serum AST and ALT value of hyperlipidemic mice treated with AR, AMR and ARD significantly reduced compared to that of HFD group. The changes of serum AST and ALT values were in agreement with the change of liver coefficient, and may provide evidence of the effects of AR, AMR and ARD on the protection of hepatocytes against liver damage induced by HFD.

Hepatic steatosis was characterized by the presence of large lipid droplets that enlarged the hepatocytes and eccentrically displaced the nucleus to the periphery (Figure 3B, b). Significant hepatic steatosis and eccentric nucleus were not observed in the AR, AMR and ARD groups. In contrast to the absence of lipid accumulation in most control livers, the livers of AR group show little macrovesicular and relatively mild lipid accumulation (Figure 3C). Furthermore, the degree of steatosis, particularly of large lipid droplets, was improved significantly in ARD-treated hyperlipidemic mice, which showed an impressive return to a normal morphology (Figure 3E). Hepatocyte injury, ballooning degeneration (Figure 3B, c) and PMN infiltration (Figure 3B, d) were seen in the liver of hyperlipidemic mice, which may lead to inflammation and steatohepatitis. However, decreases in the number of ballooning degeneration and PMN were observed in AR and ARD groups, indicating the potent anti-inflammation activity of AR and ARD.

The cholesterol biosynthesis pathway consists of more than 20 enzymes, whose expression is regulated by the SREBF-2 (18). The rate-limiting step in this pathway is the conversion of 3-hydroxy-3-methylglutaryl coenzyme A into mevalonate by HMG-CoA reductase, a key pathway of hepatic cholesterol synthesis (19). As shown in Figure 4B, the relative quantity of hepatic SREBF-2 mRNA were significantly down regulated in hyperlipidemic mice treated with AR and ARD. In addition, the expression of hepatic HMG-CoA reductase mRNA was increased in hyperlipidemic mice, and the increases were markedly improved in AR and ARD treated animals (Figure 4C). This coordinate regulation has been shown to be due to the sterol regulatory element binding factor-2 that activates transcription of genes for HMG-CoA reductase and other enzymes of the pathway for cholesterol synthesis (20). Thus, the reduction of HMG-CoA reductase expression contributing to the attenuation of hepatic cholesterol biosynthesis, and this pathway may be a major mechanism which responsible for the reduction of liver cholesterol level (21). CYP7A1 gene expression is known to be positively regulated by a cholesterol-rich diet, resulting in the production of bile acids in response to cholesterol diets (22). However, the study did not reveal significant differences on CYP7A1 gene expressions among all groups, suggesting AR and ARD could not stimulate the gene expression of hepatic enzyme CYP7A1 mRNA despite the fact that a profound inhibition effect of cholesterol accumulation in liver by AR and ARD was observed. One of the possible reasons for unaltered hepatic CYP7A1 expression is that hyperlipidemic mice maintained feed of HFD during the TCM treatments, which inhibit decreased gene expression of CYP7A1 regulated by TCMs.

TCM prescriptions are usually a decoction of more than one TCM material to obtain synergistic effect and diminish adverse reactions. The efficacy of TCMs is a characteristic of a complex mixture of chemical compounds which lead to complexity of mechanisms of pharmacological activity. ARD, a classical TCM formula, is composed of two component TCMs: AR and AMR. Previous studies both* in-vivo* and *in-vitro* have verified triterpenes as the major active ingredients in AR. The triterpenes possess potent hypolipidemic activity, as shown by improving absorption of exogenous TC and TG in diets, affecting the metabolism of endogenous TC, interfering in absorption, metabolism and excretion of cholesterol (23, 24). The hypolipidemic activity of AR suggests that AR in ARD formula may be a principle component TCM for hypolipidemic activity of ARD. The results in the present study revealed that ARD possesses a more potent hypolipidemic effect than that of AR at the equal dose. The findings suggest that AMR may serves as adjuvant one to facilitate the hypolipidemic activity of AR due to its notable pharmacological activities including hemangiectasis, anti-coagulated blood, livers protective and diuresis activities (25-28).

## Conclusions

The observed hypolipidemic effects of the ARD indicate that these plants extracts possess the potent lipid-lowering activities in hyperlipidemia male mice. The suppression of HMG-CoA reductase as well as SREBF-2 expression by AR and ARD extract may be a major mechanism leading to the attenuated biosynthesis of lipids in liver. Besides, hypolipidemic effect of ARD is more apparent than that of its component AR, suggesting a synergistic effect among AR and AMR in ARD, and confirming the rationality of ARD formula. This result is in agreement with the rationality of the Chinese herbal formulas: mutual reinforcement of the compounds (29). Further work is clearly needed to identify the bioactive components in ARD, and elucidate their molecular targets for the hypolipidemic effect. 
